# Chronic social defeat stress induces a hypoglycemic shift and depression-related behaviors, both prevented by antidiabetic drugs

**DOI:** 10.1093/ijnp/pyag046

**Published:** 2026-08-23

**Authors:** Wenran Qiu, Hirotaka Nagai, Kohei Ota, Midori Nagai, Tomoyuki Furuyashiki

**Affiliations:** Division of Pharmacology, Graduate School of Medicine, Kobe University, Kobe 650-0017, Japan; Department of Anatomy, Division of Histology and Cell Biology, School of Medicine, Jichi Medical University, Shimotsuke 329-0498, Japan; Division of Pharmacology, Graduate School of Medicine, Kobe University, Kobe 650-0017, Japan; Department of Anatomy, Division of Histology and Cell Biology, School of Medicine, Jichi Medical University, Shimotsuke 329-0498, Japan; Division of Pharmacology, Graduate School of Medicine, Kobe University, Kobe 650-0017, Japan; Division of Pharmacology, Graduate School of Medicine, Kobe University, Kobe 650-0017, Japan; Department of Anatomy, Division of Histology and Cell Biology, School of Medicine, Jichi Medical University, Shimotsuke 329-0498, Japan; Division of Pharmacology, Graduate School of Medicine, Kobe University, Kobe 650-0017, Japan; Department of Pharmacology, Graduate School of Medical and Dental Sciences, Institute of Science Tokyo, Tokyo 113-8510, Japan

**Keywords:** social defeat, mice, stress, psychological, hypoglycemia, hypoglycemic agents

## Abstract

**Objective:**

Stress-related mental illnesses, including depression, are associated with altered glucose metabolism. However, it is unclear how chronic stress alters glucose metabolism along with depression-related behaviors, and how antidiabetic drugs normalize these alterations.

**Methods:**

Using a mouse model of social defeat stress, we compared stress-induced glycemic responses among strains that differ in stress susceptibility (C57BL/6N, C57BL/6J, and BALB/c). Blood glucose was measured before stress and after the last stress exposure of acute and chronic stress. Circulating metabolic hormones and inflammatory cytokines were quantified, and depression-related behaviors (social avoidance, anhedonia-like behavior and cognitive deficits) were assessed. In C57BL/6N mice, the antidiabetic drug glyburide or liraglutide was administered 30 min before each of the 10 stress exposures, and their effects on glycemic responses and behavioral outcomes were evaluated.

**Results:**

Acute stress elevates blood glucose in the relatively stress-resilient C57BL/6N and C57BL/6J strains, but not in the more stress-susceptible BALB/c strain. After chronic stress, this stress-induced hyperglycemic response was abolished in C57BL/6N and C57BL/6J mice, whereas BALB/c mice showed stress-induced hypoglycemia. Chronic stress also produced largely coordinated changes in circulating metabolic hormones and inflammatory cytokines, with strain-dependent differences in a subset of these molecules. In C57BL/6N mice, treatment with glyburide or liraglutide, antidiabetic drugs with distinct primary mechanisms of action, before each stress exposure reduced the development of anhedonia-like behavior and cognitive deficits without affecting social avoidance, while restoring stress-induced hyperglycemia.

**Conclusions:**

These results demonstrate that chronic stress drives a hypoglycemic shift accompanied by depression-related behaviors, and that the development of both metabolic and behavioral alterations can be prevented by antidiabetic drugs with distinct primary mechanisms administered during the stress period. Thus, this study supports metabolically targeted interventions as a potential preventive strategy for stress-related mental illnesses, whereas their efficacy against an already established phenotype remains to be tested.

**Significant outcomes**

- Using a mouse model of social defeat stress, we found that acute stress elevated blood glucose in stress-resilient C57BL/6N and C57BL/6J mice but not in stress-susceptible BALB/c mice, and that chronic stress drove a hypoglycemic shift by abolishing stress-induced hyperglycemia in C57BL/6N and C57BL/6J mice while inducing stress-related hypoglycemia in BALB/c mice.
- Chronic stress also produced largely coordinated changes in circulating metabolic hormones and inflammatory cytokines, with a subset showing strain-dependent differences.
- In C57BL/6N mice, the antidiabetic drug glyburide or liraglutide, administered before each stress exposure, restored stress-induced hyperglycemia and reduced the development of anhedonia-like behavior and cognitive deficits after chronic stress.

**Limitations**

- All experiments were conducted exclusively in male mice. Given well-established sex differences in stress responsivity, glucose metabolism, and vulnerability to depression-related phenotypes, the generalizability of the present findings to females remains uncertain and warrants future investigation
- BALB/c data after chronic stress were obtained exclusively from animals that survived the stress protocol thus, the observed hypoglycemia should be interpreted as a finding in the surviving animals and should not necessarily be generalized as a characteristic response of the BALB/c strain. The high mortality also raises the possibility of survivorship bias in this group. This limitation does not affect our conclusion regarding the blunted hyperglycemic response observed in C57BL/6N and C57BL/6J mice, in which mortality during the chronic stress protocol was low.

## Introduction

A growing body of clinical evidence links major depressive disorder (MDD) to disturbances in energy metabolism. Recent clinical work has documented elevated fasting glucose in subsets of patients with MDD, and large meta-analysis supports an association between depression and insulin resistance.^1^ These converging observations align with population-level studies indicating a bidirectional relationship between depression and type 2 diabetes (T2D), with genetic and epidemiologic analyses increasingly supporting reciprocal risk and shared biology.^2^^,^^3^ Motivated by this metabolic-psychiatric interface, multiple antidiabetic agents have been evaluated in stress-based mouse models of depression, including chronic stress paradigms, where several classes show antidepressant-like effects.^4^ Especially, Glucagon-like peptide-1 (GLP-1) receptor agonists (GLP-1RAs) improve affective and cognitive readouts in a chronic unpredictable stress model and mitigate depressive-like phenotypes in chronic social defeat stress.^5–7^ Translational interest has followed: analyses of clinical trial data in obesity and early randomized studies in MDD suggest generally neutral to modest effects of GLP-1RAs on depressive symptoms, and dedicated trials explicitly targeting depressive outcomes are now underway.^8–10^

Despite the growing attention to the role of glucose metabolism in depression, how chronic stress alters glucose metabolism along with depression-related behaviors, and how antidiabetic drugs normalize these metabolic alterations remain incompletely defined. For example, chronic unpredictable mild stress can progressively induce glucose intolerance and dysregulation of appetite-related hormones,^11^ whereas other chronic stress paradigms yield insulin resistance in both hyperglycemic and normoglycemic mice.^12^ Chronic stress can even be associated with reduced blood glucose and even hypoglycemia, depending on physiological contexts and individual susceptibility.^12^ In addition, different classes of antidiabetic drugs reduce hyperglycemia via fundamentally distinct mechanisms, raising a central mechanistic question when such agents appear to improve neuropsychiatric phenotypes. Importantly, GLP-1 receptors are expressed well beyond pancreatic β cells, including in multiple brain regions and within neuronal and glial populations, supporting the plausibility of direct central actions of GLP-1RAs on neural circuits governing emotion and cognition.^13^^,^^14^ Collectively, these considerations underscore the need to examine stress-associated glucose dysregulation under carefully controlled experimental conditions and to disentangle glucose-related from glucose-unrelated pathways by directly comparing antidiabetic agents with divergent primary mechanisms.

In this study, using a mouse model of chronic social defeat stress, we investigate how acute and chronic stress alter glucose metabolism across multiple mouse strains that differ in stress susceptibility, and whether antidiabetic drugs with distinct mechanisms of action, glyburide and liraglutide, can normalize stress-related metabolic changes and depression-related behaviors. In C57BL/6N and C57BL/6J mice, acute stress induced hyperglycemia, whereas chronic stress attenuated this hyperglycemic response. By contrast, BALB/c mice, which are more stress-susceptible,^15^ did not exhibit acute stress-induced hyperglycemia and instead developed chronic stress-induced hypoglycemia. In C57BL/6N mice, treatment with either glyburide or liraglutide restored stress-induced hyperglycemia and ameliorated anhedonia-like behavior and cognitive deficits after chronic stress. These findings support a strain-dependent link between chronic stress-induced glucose dysregulation and behavioral impairments, and suggest that metabolically targeted interventions, including mechanistically distinct antidiabetic agents, may alleviate chronic stress-related phenotypes.

## Materials and methods

### Animals

Male C57BL/6N, C57BL/6J, and BALB/c mice (7-9 weeks old), as well as male Institute of Cancer Research (ICR) mice retired from breeding, were obtained from Japan SLC (Shizuoka, Japan). Animals were housed in a controlled environment under stable temperature and humidity conditions with a 12-h light/12-h dark cycle for at least one week before experiments. Food and water were provided *ad libitum*. Mice were initially group-housed (4-5 per cage) and subsequently transitioned to single housing prior to behavioral experiments.

### Drug administration

Glyburide (sold under the name Glibenclamide; S1716, Selleck Chemicals, Houston, TX, USA) was dissolved in saline containing 2% DMSO and 2% Tween-80, warmed to 37°C, mixed thoroughly, and administered intraperitoneally at a dose of 10 mg/kg/day, 30 min before each exposure to social defeat stress, as described below.^16^ Liraglutide (S8256, Selleck Chemicals) was dissolved in saline containing 2% DMSO and 2% Tween-80 and administered intraperitoneally at 20 nmol/kg/day, 30 min prior to each stress exposure.^17^ Drug doses were determined according to previous studies.^16^^,^^17^ Saline containing 2% DMSO and 2% Tween-80 without either drug was administered intraperitoneally with the same volume (10 μL/g body weight) and served as the vehicle control.

### Social defeat stress

Social defeat stress was performed as previously described with minor modifications.^18^^,^^19^ Briefly, male ICR mice were screened for aggressiveness toward a male C57BL/6N mouse based on attack latency and number of attacks during 3-min daily sessions for three consecutive days. Only mice exhibiting consistent aggression were used as resident aggressors. After 1 week of single housing, experimental C57BL/6N, C57BL/6J, or BALB/c mice (intruders) were subjected to a daily 10-min defeat episode for 10 consecutive days. During each session, an intruder mouse was introduced into the home cage of a resident ICR mouse, allowing direct physical interaction. To minimize variability in aggressor behavior, experimental mice were exposed to a different aggressive ICR mouse each day. Naïve mice served as unstressed controls and were left undisturbed in their home cages. Throughout the chronic stress protocol, all animals were observed daily for their general condition, including spontaneous activity, posture, coat condition, responsiveness to handling, apparent food and water consumption, and signs of respiratory or other physical distress. Humane endpoints were substantial or progressive deterioration in general condition, including marked loss of body weight, persistent inability or reluctance to eat or drink, pronounced reduction in activity or impaired mobility, persistent abnormal posture, severe dehydration, marked respiratory distress, or markedly reduced responsiveness; animals meeting these criteria were to be withdrawn from the experiment and euthanized. Despite this monitoring, some mice, especially those of BALB/c background, subjected to chronic stress died without clear preceding clinical signs that would have met or predicted these humane endpoints, indicating that the deterioration leading to death could not always be anticipated from routine daily observation. Whereas most C57BL/6N and C57BL/6J mice survived chronic stress (C57BL/6N: 77 out of 85; C57BL/6J: 20 out of 22), survival was substantially lower in BALB/c mice (19 out of 42), reducing the numbers of BALB/c mice analyzed after chronic stress. This magnitude of mortality in BALB/c mice was not anticipated when the study was designed and when the animal experimental protocol was approved, and it was substantially greater than that observed in the 2 C57BL/6 substrains, suggesting that BALB/c mice were more vulnerable to this chronic stress paradigm than originally expected.

### Social interaction test

The social interaction test was performed as previously described.^18–20^ On Day 0 (1 day before the first stress exposure), mice were placed in a behavioral chamber (30 × 40 cm) containing an empty mesh cage (12 × 5.5 cm) positioned at one end under 3-lux red light illumination and allowed to explore freely for 150 s. Social interaction testing was performed after the first and tenth stress exposure with a novel ICR mouse placed inside the mesh cage. Behavior was video-recorded and analyzed using SMART video-tracking software (PanLab Harvard Apparatus, Holliston, MA, USA). The interaction zone was defined as the area around the mesh cage, and the avoidance zone as the area at the opposite end of the chamber. The percentage of time spent in the avoidance zone was used as an index of social avoidance. For post-hoc analyses, C57BL/6N mice subjected to chronic stress were further classified as stress-susceptible (spending more than 50% of the time in the avoidance zone) or stress-resilient (spending less than 50% of the time in the avoidance zone).

### Novel object recognition test

The novel object recognition test was performed as previously described with minor modifications.^21^ The test consisted of 2 sessions conducted in a 30 × 40 cm chamber. During the habituation session, 2 identical LEGO blocks (3 × 3 × 8 cm) were placed in the chamber, and mice were allowed to explore the objects for 600 s. Four hours later, a test session was conducted with one familiar LEGO block and one novel object (sand-filled flask; 5.5 × 2.5 × 9.5 cm). Mice explored the objects freely for 600 s or until total object exploration time reached 20 s, whichever occurred first. Novel object preference scores were calculated as the percentage of time exploring the novel object relative to total object exploration time. Mice that didn’t explore either object (total exploration time < 1 s) were excluded.

### Female urine sniffing test

The female urine sniffing test was performed as previously described.^22^ After habituation in the testing room under 3-lux white light for 30 min, mice were habituated for 60 min to a cotton swab soaked with 25 μL distilled water and attached to the inner wall of the home cage. Two 300-s sessions were conducted and video-recorded. In the first session, a new swab soaked with 25 μL distilled water was presented; in the second session, a swab soaked with 25 μL freshly collected urine from female C57BL/6N mice (10-16 weeks old) was presented. Sniffing time directed at the swab tip (excluding biting) was quantified as an index of reward preference. Mice that failed to detect the swab (maximum continuous sniffing <2 s) were excluded.

### Blood glucose measurement

Blood glucose levels were measured at 4 time points: before, immediately, 30 min, and 60 min after the first and tenth stress exposure. Blood was collected from the tail vein under restraint, and glucose concentrations were measured using a mouse glucose meter (LABGluco, ForaCare Japan Co., Ltd., Tokyo, Japan; measurement range, 20-600 mg/dL). In experiments with antidiabetic drugs, which were administered 30 min before the first and tenth stress exposures, blood glucose levels were measured at baseline (immediately before drug administration), and immediately, 30 min, and 60 min after stress exposure.

### Measurement of metabolic hormones and inflammatory cytokines

To minimize the effects of feeding on metabolic hormones, mice were food-deprived starting between zeitgeber time (ZT) 1 and ZT2. After a 6-h resting period, mice were deeply anesthetized via intraperitoneal injection of sodium pentobarbital (100 mg/kg; Nacalai Tesque, Kyoto, Japan), and 200 μL of blood was slowly collected from the left ventricle using a 21-gauge needle. The collected blood was immediately transferred to a microcentrifuge tube and supplemented with 2 μL of a DPP4 inhibitor (2.5 mM stock; Merck KGaA, Darmstadt, Germany) and 2 μL of phenylmethylsulfonyl fluoride (200 mM stock in isopropanol; 06297-31, Nacalai Tesque). Blood samples were allowed to clot for 30 min at room temperature and then centrifuged at 1000 × g for 10 min at 4°C. The supernatant was collected as serum and aliquoted (50 μL per tube) for storage at -80°C until use. Serum levels of metabolic hormones and inflammatory cytokines were measured using the Milliplex Mouse Metabolic Hormone Expanded Panel Kit (MMHE-44K; Merck) on a MAGPIX system (Luminex Corporation, Austin, TX, USA). Concentrations below the detection limit were assigned a value equal to half of the lower detection limit specified in the manufacturer’s protocol. Principle component analysis was performed using R.

### Statistical analysis

Data are shown as means ± standard error of means (SEM). Statistical analyses were performed using Prism 10.6 software (GraphPad Software, San Diego, CA, USA). *P* values less than .05 were considered statistically significant. Comparisons between two groups were analyzed using unpaired *t*-tests. Comparisons among more than 2 groups with 2 independent variables were analyzed using two-way analysis of variance (ANOVA) followed by Tukey’s or Holm-Sidak’s multiple comparison tests, as appropriate. For repeated-measures datasets with missing values due to mortality, a mixed-effects model (restricted maximum likelihood) was used. Comparisons among more than 2 groups with a single independent variable were analyzed by one-way ANOVA followed by Holm-Sidak’s multiple comparison tests. Correlations were assessed using Pearson’s correlation test. Outliers were identified using Grubbs’ test and excluded where indicated.

## Results

### Chronic stress disrupts blood glucose regulation and metabolic hormones alongside depression-related behaviors

**Figure 1:**
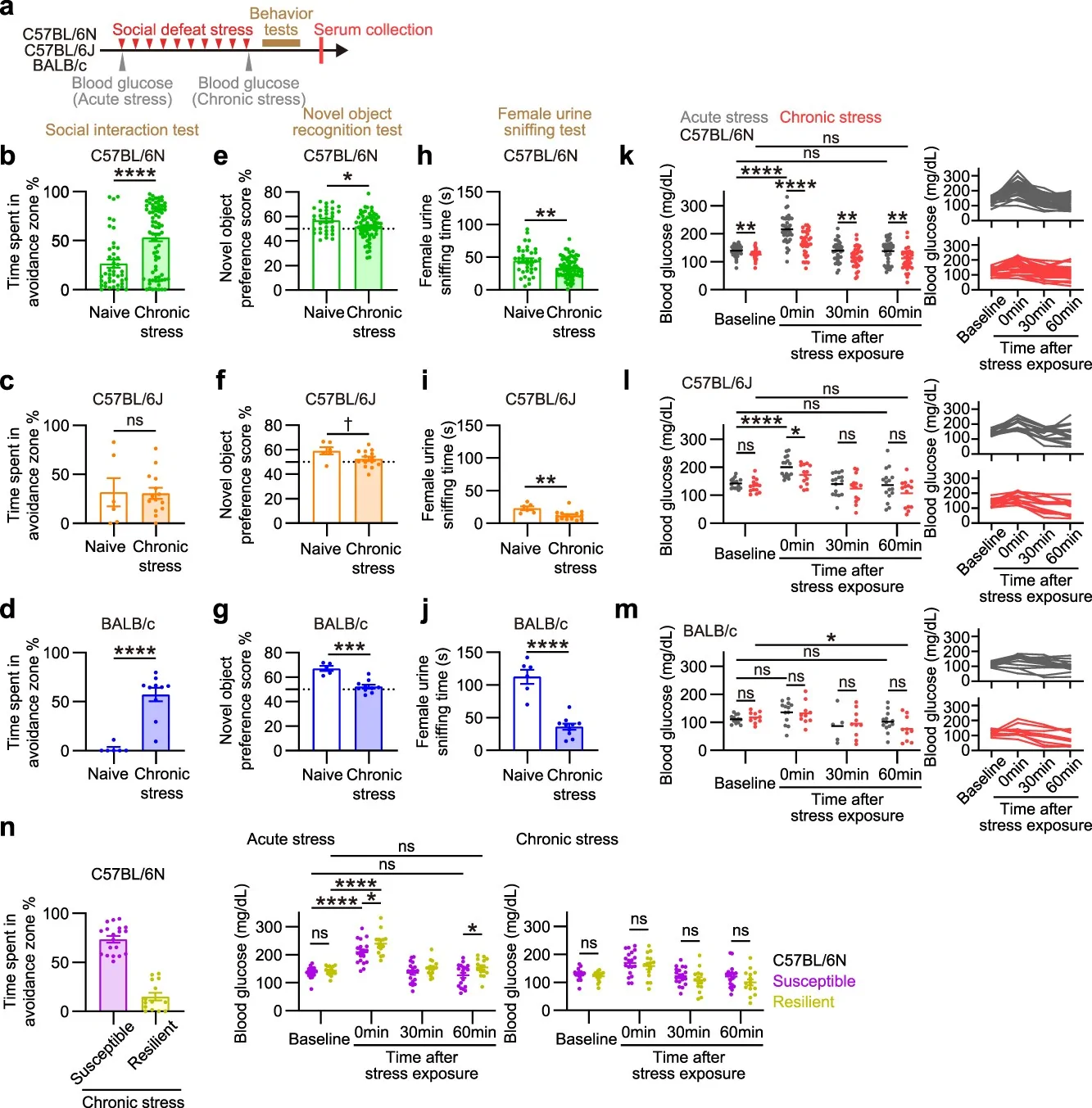
Chronic stress causes multiple behavioral alterations in a strain-dependent manner. (a) Experimental schedule. (b-j) Levels of social avoidance (b-d), reduced recognition and preference to a novel object (e-g), and reduced preference to female urine (h-j) in C57BL/6N (b, e, h), C57BL/6J (c, f, i), and BALB/c (d, g, j) mice without (Naive) or with chronic social defeat stress. (k-m) Blood glucose levels measured at baseline and immediately (0 min), 30 min, or 60 min after the last stress exposure under acute or chronic stress conditions in C57BL/6N (k), C57BL/6J (l), and BALB/c (m) mice, shown as group data with individual points (left) and as individual animal time-course (spaghetti) plots for acute (top) and chronic (bottom) stress conditions (right). (n) C57BL/6N mice were stratified into stress-susceptible and stress-resilient subgroups based on time spent in the avoidance zone after chronic stress (left). Blood glucose levels in susceptible and resilient mice were measured at baseline and immediately (0 min), 30 min, or 60 min after the first (acute stress, middle) or 10th (chronic stress, right) stress exposure. Data are presented as mean ± SEM. †*P* < .1, ^*^P < .05, ^**^P < .01, ^***^P < .001, ^****^P < .0001, and ns, not significant for unpaired two-tailed Student’s *t*-tests (b-j) or Tukey’s multiple comparisons following two-way ANOVA (k-n). In behavioral analyses (b-j), *n* = 44 and 76 for C57BL/6N mice in naive and chronic stress conditions, respectively; *n* = 6 and 14 for C57BL/6J mice in naive and chronic stress conditions, respectively; *n* = 6 and 10 for BALB/c mice in naive and chronic stress conditions, respectively. In blood glucose analyses (k-m), *n* = 38 and 34 for C57BL/6N mice after acute and chronic stress, respectively; *n* = 16 and 14 for C57BL/6J mice after acute and chronic stress, respectively; *n* = 13 and 10 for BALB/c mice after acute and chronic stress, respectively. In (*n*), *n* = 19 and 15 for susceptible and resilient C57BL/6N mice, respectively.

To investigate the effects of acute and chronic stress on blood glucose levels and the development of depression-related behaviors, 3 mouse strains (C57BL/6N, C57BL/6J, and BALB/c) were subjected to chronic social defeat stress (Figure 1a). Depression-related behaviors were assessed by measuring social interest toward a novel target, preference for a novel object, and preference for female urine, reflecting social behavior, object memory and preference, and motivation toward hedonic stimuli, respectively (Figure 1b-j). C57BL/6J mice exhibited impaired novel object recognition and reduced preference for female urine, while showing minimal social avoidance (Figure 1c,f,i). Similarly, C57BL/6N mice displayed impairments in novel object recognition and female urine preference; however, they exhibited higher levels of social avoidance with substantial individual variability, indicating the presence of stress-resilient individuals (Figure 1b,e,h). In contrast, BALB/c mice showed marked impairments in novel object recognition and female urine preference, with a more pronounced reduction in female urine preference due to their higher baseline preference compared with C57BL/6J and C57BL/6N mice (Figure 1d,g,j). Moreover, BALB/c mice demonstrated significantly greater social avoidance, with only a small proportion of stress-resilient individuals (Figure 1d). Collectively, these results indicate that chronic stress causes multiple behavioral alterations in a strain-dependent manner, with BALB/c mice showing greater susceptibility to chronic stress compared with C57BL/6J and C57BL/6N mice, consistent with previous reports.

In addition to behavioral analyses, blood glucose levels were measured following the first (acute) and tenth (chronic) stress exposures (Figure 1a). In both C57BL/6N and C57BL/6J mice, acute stress elicited a pronounced transient hyperglycemic response, which returned to baseline within 60 min (Figure 1k,l). This stress-induced hyperglycemic response was markedly attenuated after chronic stress exposure. In contrast, BALB/c mice did not exhibit a hyperglycemic response to either acute or chronic stress (Figure 1m). Instead, a hypoglycemic response to stress emerged following chronic stress exposure and was gradually induced over a 60-min period after stress. Individual time-course plots revealed substantial within-strain variability in these glycemic responses, particularly among C57BL/6N mice after chronic stress (Figure 1k-m). To examine whether this variability reflects the stress-susceptible phenotype, C57BL/6N mice were further stratified into stress-susceptible and stress-resilient subgroups based on social avoidance levels after chronic stress (Figure 1n). Susceptible mice showed a significantly weaker hyperglycemic response than resilient mice immediately (0 min) after the first, acute stress exposure, and this difference persisted at 60 min (Figure 1n). In contrast, the 2 subgroups showed comparable glycemic responses at all time points after chronic stress (Figure 1n). These results indicate that the magnitude of the acute hyperglycemic response, which differed between susceptible and resilient mice, may serve as an early trait associated with subsequent susceptibility. However, this initial difference was no longer apparent after chronic stress, when both subgroups converged to a similarly blunted glycemic response, indicating that the hypoglycemic shift after chronic stress is not restricted to the stress-susceptible phenotype. These findings indicate that chronic stress differentially dysregulates blood glucose levels across mouse strains, with lower glucose levels after chronic stress observed in strains with greater stress susceptibility, whereas, within the C57BL/6N strain, the acute hyperglycemic response is inversely associated with individual differences in stress susceptibility.

**Figure 2:**
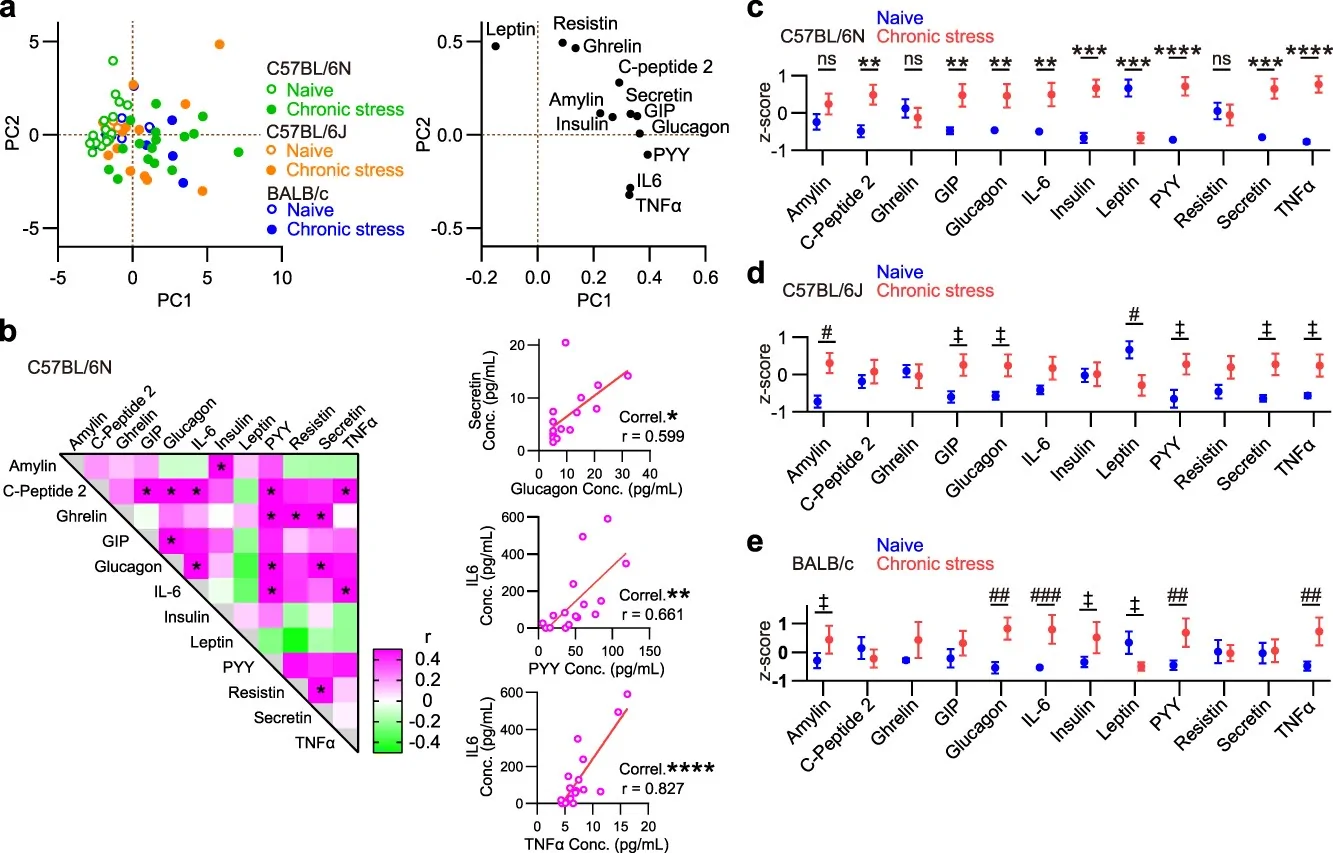
Chronic stress induces largely coordinated changes in circulating metabolic hormones and inflammatory cytokines across different mouse strains. (a) Principal component analysis of z-scored levels of circulating metabolic hormones and inflammatory cytokines in C57BL/6N, C57BL/6J, and BALB/c mice without (Naive) or with chronic social defeat stress. Values of principal component 1 (PC1) and PC2 are plotted for individual mice (left). Loadings of PC1 and PC2 are plotted for each metabolic hormone and inflammatory cytokine (right). (b) Correlation matrix (left) and representative correlations (right) among z-scored levels of circulating metabolic hormones and inflammatory cytokines in C57BL/6N with chronic social defeat stress. Pearson’s correlation coefficients (r) are shown. Correl., correlation. (c-e) Z-scored levels of metabolic hormones and inflammatory cytokines in C57BL/6N (c), C57BL/6J (d), and BALB/c (e) mice without (Naive) or with chronic social defeat stress. Data are presented as mean ± SEM. ^*^P < .05, ^**^P < .01, ^***^P < .001, ^****^P < .0001, and ns, not significant for Pearson’s correlation (b) and Holm-Sidak’s multiple comparison tests following two-way ANOVA (c). ‡*P* < .1 and #*P* < .05, ##*P* < .01, ###*P* < .001 for planned comparisons using unpaired *t*-tests for stress-affected hormones (d, e). *n* = 16 and 16 for C57BL/6N without (Naive) or with chronic social defeat stress; *n* = 6 and 14 for C57BL/6J mice without (Naive) or with chronic social defeat stress; *n* = 10 and 5 for BALB/c mice without (Naive) or with chronic social defeat stress.

We next investigated the effects of chronic stress on circulating metabolic hormones and inflammatory cytokines that regulate blood glucose levels. Principal component analysis revealed that chronic stress induced similar overall changes in the profiles of metabolic hormones and cytokines across mouse strains, as indicated by a consistent increase in PC1 values (Figure 2a). Correlation matrix analyses demonstrated positive correlations among metabolic hormones (eg, glucagon and secretin), among inflammatory cytokines (eg, TNF-α and IL-6), and between metabolic hormones and inflammatory cytokines (eg, PYY and IL-6), suggesting that chronic stress elicits coordinated alterations in these circulating factors (Figure 2b). Consistent with these global changes, chronic stress significantly increased the levels of C-Peptide 2, GIP, glucagon, IL-6, insulin, PYY, secretin, and TNF-α, while decreasing leptin levels in C57BL/6N mice (Figure 2c). Similar directional changes were observed in C57BL/6J and BALB/c mice, albeit with limited statistical significance attributable to smaller sample sizes (Figure 2d,e). However, several hormones showed strain-dependent differences. Secretin, a hormone known to support synaptic integrity and to influence social and cognitive behaviors,^23^ was upregulated in C57BL/6J and C57BL/6N mice, but not in BALB/c mice. In contrast, IL-6, a cytokine implicated in chronic stress-induced depression-related behaviors,^24^ was robustly upregulated in BALB/c and C57BL/6N mice, but showed a weaker response in C57BL/6J mice, which are less stress-susceptible than the other two strains (Figure 2c-e). Together, these results indicate that chronic stress induces largely coordinated changes in circulating metabolic hormones and inflammatory cytokines, with strain-dependent differences in a subset of these molecules.

To determine whether strain-dependent differences in feeding behavior or body weight could account for these glycemic and endocrine differences, we monitored 3-h dark-phase food intake and daily body weight throughout the 10-day chronic stress protocol in an independent cohort of C57BL/6N and BALB/c mice (Supplementary Figure S1). Food intake did not differ between strains and was not altered by chronic stress in either strain. Body weight remained stable in C57BL/6N mice, whereas BALB/c mice as a group showed a marked overall decrease. However, this decrease was driven primarily by animals that subsequently died during the stress protocol, and BALB/c survivors showed only a mild, non-significant downward trend. These results indicate that the strain-dependent glycemic and endocrine phenotypes described above are not attributable to differences in feeding behavior or a general decline in health status, but instead reflect a more direct metabolic consequence of chronic stress.

### Administration of antidiabetic drugs during stress exposure prevented the development of cognitive and hedonic deficits and partially normalizes metabolic profiles

**Figure 3:**
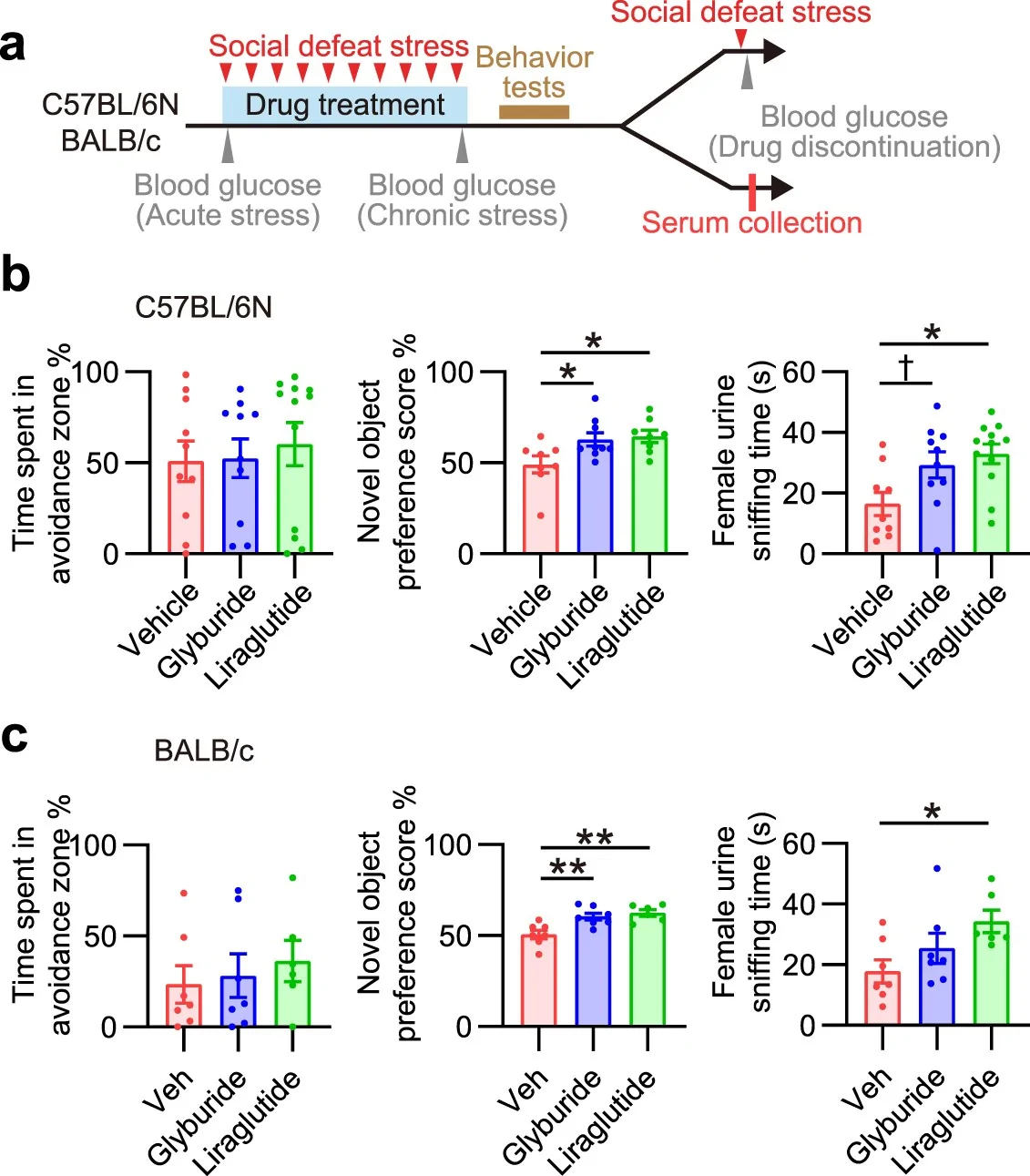
Antidiabetic drugs mitigate chronic stress-induced behavioral alterations. (a) Experimental schedule. (b and c) Levels of social avoidance, reduced recognition and preference to a novel object, and reduced preference to female urine in C57BL/6N (b) and BALB/c (c) mice following chronic social defeat stress, treated with vehicle, glyburide, or liraglutide 30 min before each of the ten stress exposures, with treatment discontinued during behavioral testing. Data are presented as mean ± SEM. †*P* < .1, ^*^P < .05, ^**^P < .01 and ns, not significant for Holm-Sidak’s multiple comparison tests following one-way ANOVA. *n* = 15, 13, and 14 for C57BL/6N mice treated with vehicle, glyburide, and liraglutide, respectively; *n* = 7, 7, and 6 for BALB/c treated with vehicle, glyburide, and liraglutide, respectively.

Hyperglycemia induced by acute stress and its suppression following chronic stress led us to hypothesize that glucose dysregulation is involved in the pathology of chronic stress and that this pathology may be mitigated by antidiabetic drugs. Glyburide is a sulfonylurea drug that inhibits ATP-sensitive K^+^ channels, thereby promoting insulin secretion.^25^ In contrast, liraglutide is a GLP-1 agonist that acts on its cognate Gs-coupled receptor and promotes insulin secretion via a distinct mechanism.^26^ We therefore reasoned that these two drugs exert common effects on abnormalities arising from hyperglycemia per se during stress exposure. Treatment with both drugs 30 min prior to each stress episode, which was discontinued during behavioral testing, reduced the development of chronic stress-induced cognitive decline and anhedonia-like behavior in C57BL/6N mice, without affecting social avoidance (Figure 3a,b). Similar behavioral effects were observed in BALB/c mice (Figure 3c).

**Figure 4:**
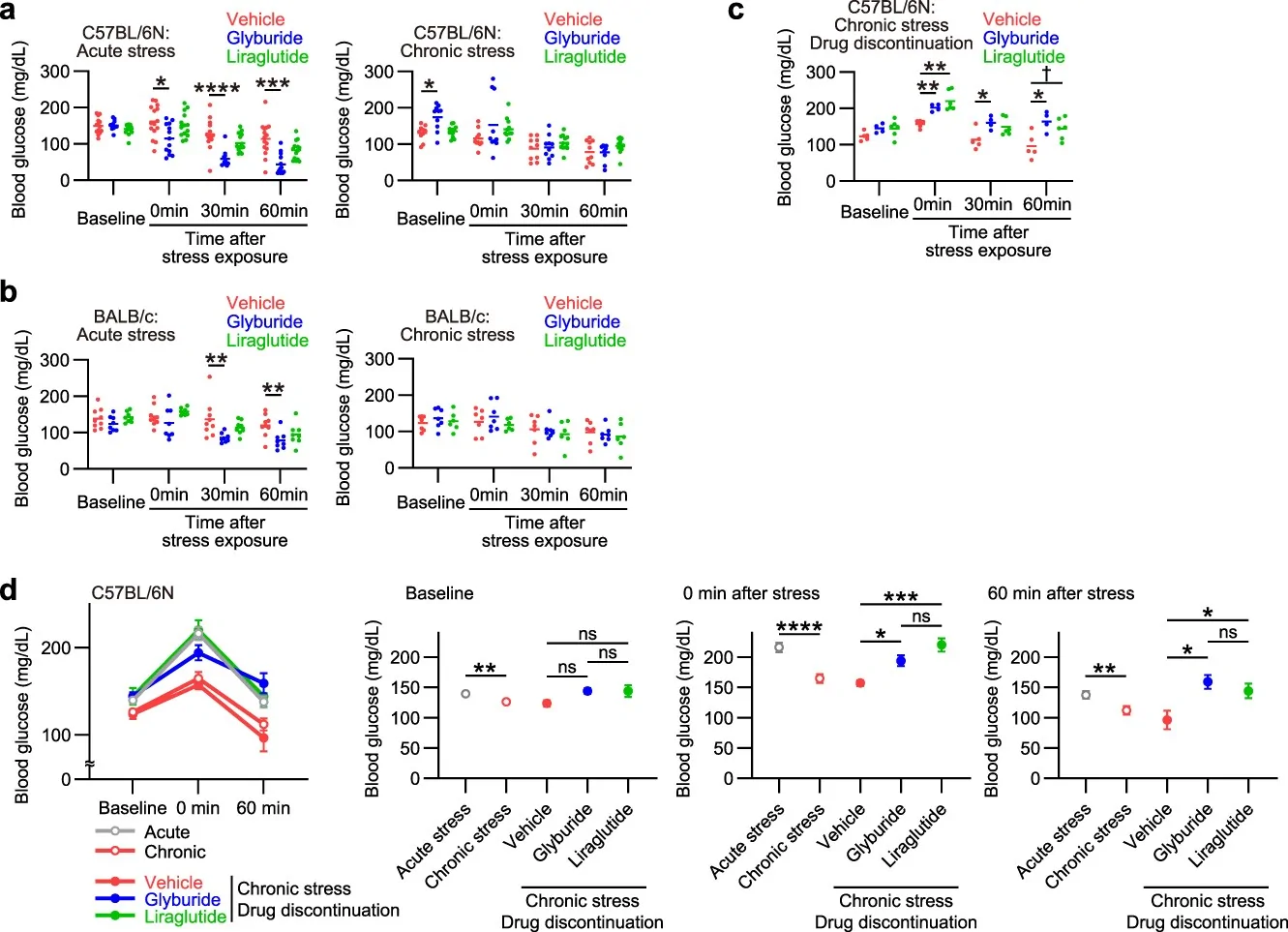
Antidiabetic drugs restore stress-induced hyperglycemia after chronic stress. (a-c) Blood glucose levels measured at baseline and immediately (0 min), 30 min, or 60 min after the last stress exposure under acute or chronic stress conditions in C57BL/6N (a) and BALB/c (b) mice, treated with vehicle, glyburide, or liraglutide, and in C57BL/6N mice after chronic stress with subsequent drug discontinuation (c). Baselines were obtained without drug administration or stress exposure. Mice received intraperitoneal injections of vehicle, glyburide, or liraglutide 30 min prior to stress exposure. An experimental schedule is shown in Figure 3a. (d) Blood glucose levels in C57BL/6N mice treated with vehicle, glyburide, or liraglutide and after drug discontinuation, derived from (c). Data for acute and chronic stress without drug treatments are derived from Figure 1k. Data are presented as mean ± SEM. †*P* < .1, ^*^P < .05, ^**^P < .01, ^***^P < .001, ^****^P < .0001, and ns, not significant for Tukey’s multiple-comparisons test following two-way ANOVA (a-c), unpaired *t*-tests (d, between acute and chronic stress), and Holm-Sidak’s multiple-comparisons following one-way ANOVA (d, among drug conditions). *n* = 15, 13, and 14 for C57BL/6N mice treated with vehicle, glyburide, and liraglutide, respectively (a); *n* = 7, 7, and 6 for BALB/c mice treated with vehicle, glyburide, and liraglutide, respectively (b); *n* = 5, 4, and 6 for C57BL/6 mice treated with vehicle, glyburide, and liraglutide, respectively, and after drug discontinuation (c).

Despite these consistent behavioral effects of the two antidiabetic drugs, their immediate effects on glucose responses to acute and chronic stress differed. Treatment with glyburide, but not liraglutide, administered 30 min prior to each stress exposure reduced glucose levels after acute stress and increased glucose levels 24 h after the final drug treatment and the last (ninth) stress exposure during chronic stress in C57BL/6N mice (Figure 4a). A similar effect was observed in BALB/c mice (Figure 4b). To directly test whether these drugs engage their expected insulinotropic targets, we measured serum insulin 60 min after a single injection of vehicle, glyburide, or liraglutide. In naive C57BL/6N mice, glyburide significantly increased insulin relative to vehicle, whereas liraglutide did not; in BALB/c mice, glyburide showed a trend toward increased insulin, whereas liraglutide showed no evidence of increased insulin in either naive or chronically stressed animals (Supplementary Figure S2). However, after three days of behavioral testing, the drug-induced effects on baseline glucose levels had disappeared. At this point, as expected, vehicle-treated mice showed a suppressed hyperglycemic response to an additional stress exposure following chronic stress. In contrast, mice that had been treated with both antidiabetic drugs during the chronic stress period exhibited a hyperglycemic response comparable to that observed after acute stress (Figure 4c).

Collectively, these findings demonstrate that, at least in C57BL/6N mice, treatment with both glyburide or liraglutide during the stress period restored stress-induced hyperglycemia and reduced the development of anhedonia-like behavior and cognitive deficits after chronic stress.

**Figure 5:**
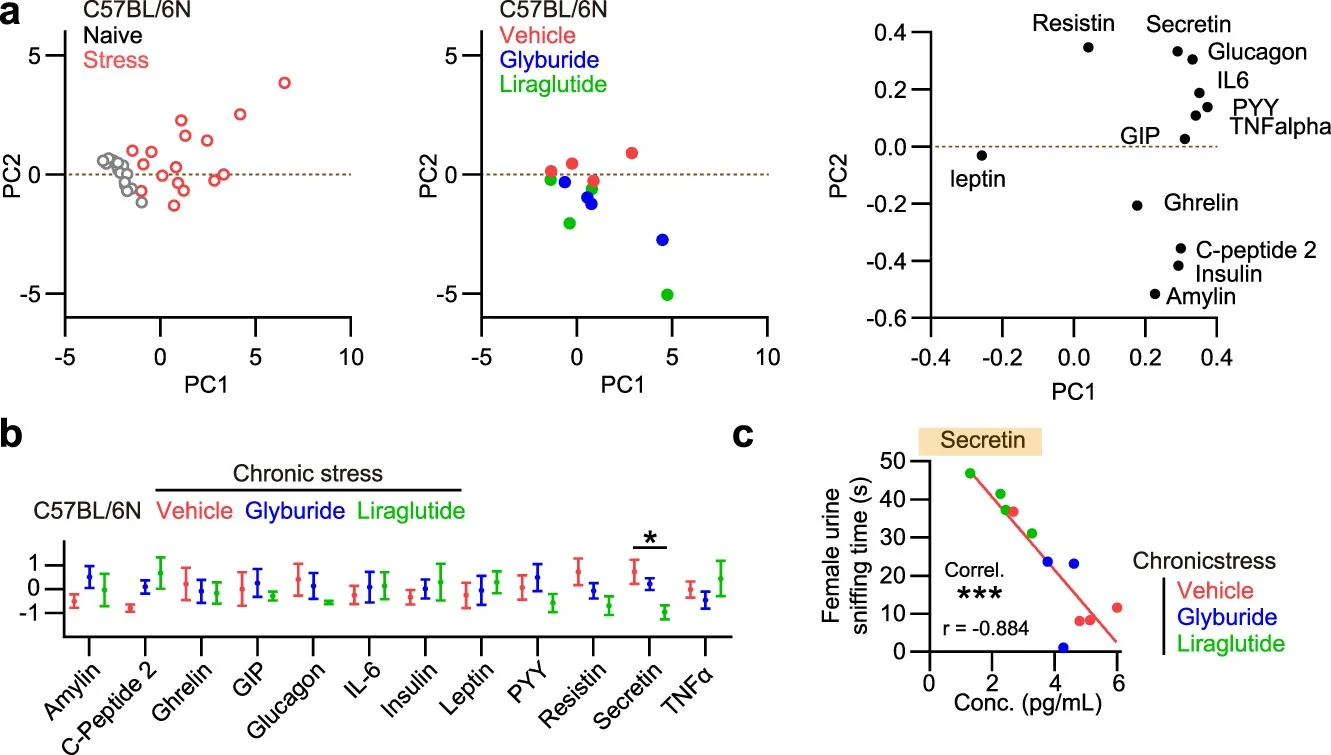
Antidiabetic drugs alter only a small subset of metabolic hormones after chronic stress. (a) Principal component analysis of z-scored levels of circulating metabolic hormones and inflammatory cytokines in C57BL/6N mice without (Naive) or with chronic social defeat stress and without drug treatment (left) and in C57BL/6N mice following chronic stress with vehicle, glyburide, and liraglutide treatment and after drug discontinuation (middle). Values of principal component 1 (PC1) and PC2 are plotted for individual mice (left). Loadings of PC1 and PC2 are plotted for each metabolic hormone and inflammatory cytokine (right). Data of C57BL/6 mice without drug treatment are derived from Figure 2a. An experimental schedule is shown in Figure 3a. (b) Z-scored levels of metabolic hormones and inflammatory cytokines in C57BL/6N following chronic social defeat stress with vehicle, glyburide, and liraglutide treatment. (c) Representative correlation between metabolic hormone levels and chronic stress-induced behavioral changes in C57BL/6N mice. Pearson’s correlation coefficients (r) are shown. The correlation was calculated across animals pooled from the vehicle, glyburide, and liraglutide groups; because the treatment groups differ in both variables, between-group differences contribute to the coefficient. Correl., correlation. Data are presented as mean ± SEM. ^*^P < .05, ^***^P < .001 for planned comparisons for stress-affected hormones using Holm-Sidak’s multiple comparison tests following one-way ANOVA (b) and for Pearson’s correlation (c). *n* = 5, 4, and 6 for C57BL/6N mice treated with vehicle, glyburide, and liraglutide, respectively.

We also examined the effects of glyburide and liraglutide on metabolic hormone profiles after chronic stress. The effects of these drugs varied among metabolic hormones. Principal component analysis revealed that glyburide and liraglutide did not simply reverse PC1 values, which represent the overall effects of chronic stress, but instead decreased only PC2 values, which reflect differential effects among metabolic hormones (Figure 5a). Supporting this notion, glyburide and liraglutide did not affect most stress-affected hormones, except secretin, which was decreased by liraglutide in a manner negatively correlated with the female urine sniffing time (Figure 5b and c). This correlation was calculated across animals pooled from the vehicle, glyburide, and liraglutide groups; because the treatment groups themselves differ in both secretin levels and sniffing time, between-group differences contribute to the coefficient, which therefore describes an association across the pooled cohort rather than a within-group relationship.

## Discussion

Using a mouse model of chronic social defeat stress, we found that acute stress elevates blood glucose selectively in the relatively stress-resilient C57BL/6N and C57BL/6J strains, whereas chronic stress abolishes this hyperglycemic response in these strains and instead induces hypoglycemia under stress in the more stress-susceptible BALB/c strain. This hypoglycemic shift after chronic stress was accompanied by shared changes in metabolic hormones across the strains examined, indicating a common endocrine-metabolic signature. Moreover, in C57BL/6N mice, treatment with glyburide or liraglutide, antidiabetic drugs with distinct primary mechanisms of action, before each of the 10 stress exposures reduced the development of anhedonia-like behavior and cognitive deficits, while restoring stress-induced hyperglycemia. Collectively, these results support a strain-dependent link between chronic stress-induced glucose dysregulation and behavioral impairments and point to metabolically targeted interventions as a potential strategy for preventing the development of chronic stress phenotypes.

Acute stress-induced hyperglycemia is often considered adaptive as it mobilizes energy to meet increased metabolic demand during threat. This hyperglycemia is mediated by a brain-liver pathway in which the medial amygdala projects to the ventromedial hypothalamus, drives sympathetic activation, and promotes hepatic gluconeogenesis.^27^ The present study shows that expression of this response is strain dependent, suggesting that regulation of the brain-liver pathway differs across genetic backgrounds. Such differences are plausible because stress susceptibility varies genetically and across individuals and is reflected in multiple brain regions, including the prefrontal cortex, hippocampus, and midbrain dopaminergic regions, that project to and regulate subcortical areas such as the medial amygdala.^28–30^ Supporting strain differences in stress susceptibility, acute restraint stress elicits higher medial prefrontal c-Fos immunoreactivity in C57BL/6 mice than in BALB/c mice.^31^ Peripheral metabolism may also contribute. Feeding suppresses hepatic gluconeogenic programs, and downregulates the key gluconeogenic enzyme Pck1 to a greater extent in BALB/c than in C57BL/6 mice,^32^ whereas C57BL/6 mice are more prone to diet-induced insulin resistance and obesity.^33^ Consistently, chronic stress increases body weight in C57BL/6 mice but decreases it in BALB/c mice,^34^ a pattern we also observed in our own cohort. Thus, while stress-induced hyperglycemia may be beneficial in the short term, it may also elevate long-term risk of metabolic disorders such as diabetes and obesity.

In contrast to the strain differences in acute hyperglycemia, the hypoglycemic shift after chronic stress appeared across strains, suggesting convergent mechanisms. Chronic stress induces dendritic and synaptic shrinkage and reduces neuronal responsiveness in the medial prefrontal cortex, changes linked to depression-related behaviors.^35^ Because the medial prefrontal cortex provides excitatory input to the medial amygdala, stress-induced cortical atrophy could weaken amygdala-driven hyperglycemic output, biasing the system toward lower glucose after chronic stress. In addition, chronic stress elicits metabolic and inflammatory changes in the liver, leading to the release of soluble factors that act on brain cells and promote depression-related behaviors.^36^ Since chronic stress induced largely coordinated changes in circulating metabolic hormones and inflammatory cytokines, which influence blood glucose, an interacting hormone-inflammation network may also help drive chronic stress-induced metabolic and behavioral phenotype.

Among the endocrine changes observed, chronic stress increased circulating glucagon, IL-6, TNF-α, and PYY across all 3 strains regardless of differing stress susceptibility, suggesting that these factors reflect the occurrence of stress itself and may contribute to the depression-related behavioral changes shared across strains. By contrast, the accompanying changes in β-cell-derived hormones and secretin were markedly strain-dependent. In C57BL/6N mice, both insulin and C-peptide 2, which is co-secreted with insulin in an equimolar ratio during proinsulin processing, were significantly increased, consistent with a genuine increase in β-cell secretory output. In C57BL/6J mice, neither insulin nor C-peptide 2 changed, whereas amylin, another β-cell product, was significantly increased, suggesting a secretory pattern selective for amylin in this strain. In BALB/c mice, insulin showed a trend toward an increase that was not accompanied by a corresponding change in C-peptide 2, raising the possibility that this apparent rise reflects altered insulin clearance or degradation rather than increased pancreatic secretion; amylin showed a parallel trend increase in this strain. This reciprocal, strain-dependent dissociation among insulin, C-peptide 2, and amylin indicates that chronic stress does not engage a single, uniform β-cell secretory program, but instead differentially affects the release, processing, or clearance of these hormones depending on genetic background. Secretin showed a related strain-dependent pattern: it was upregulated in the relatively stress-resilient C57BL/6N and C57BL/6J strains but not in the more stress-susceptible BALB/c strain, raising the possibility that secretin reflects an active resilience-related process rather than stress exposure per se, consistent with its reported role in supporting synaptic integrity.^23^ Notably, liraglutide reduced secretin in a manner correlated with improved female urine sniffing, an apparently paradoxical result if secretin elevation were simply protective; we speculate that secretin elevation instead reflects a compensatory response to unresolved stress, such that an effective treatment acting through an independent, more upstream mechanism could reduce the need for this response. This interpretation remains correlative, and pharmacological manipulation of secretin signaling during chronic stress will be needed to establish causality. Together, these findings suggest that chronic stress engages both common and strain-specific endocrine adaptations. While BALB/c mice lack the secretin response observed in the more resilient C57BL/6 substrains, they nevertheless develop similar behavioral impairments, indicating that multiple endocrine pathways can converge on common neural mechanisms underlying stress-induced anhedonia and cognitive dysfunction. The precise contribution of each circulating hormone to these behavioral phenotypes will require future mechanistic studies.

Both antidiabetic drugs attenuated the development of depression-related behaviors and preserved the stress-induced hyperglycemic response after chronic stress. What the 2 treatments shared was not their immediate glycemic effect but the preservation of glycemic responsiveness after chronic stress: glyburide attenuated the acute stress-induced glycemic response whereas liraglutide did not, and glyburide but not liraglutide detectably raised circulating insulin. This dissociation is expected from their pharmacology, because sulfonylureas promote insulin release in a relatively glucose-independent manner, whereas GLP-1 receptor agonists enhance glucose-dependent, physiological insulin secretion. A shared insulinotropic mechanism was therefore not demonstrated at the doses used. Yet both restored the hyperglycemic response after chronic stress and both improved behavior. These findings suggest that the blunted stress-induced hyperglycemic response reflects a stress-induced impairment of the neuroendocrine control of glucose that develops in parallel with behavioral pathology, rather than a compensatory adaptation to preceding hyperglycemia, and that the 2 drugs act during the stress period through a shared, glucose-related mechanism preserving this responsiveness after chronic stress. Consistent with a specific effect on glucose regulation, antidiabetic treatment spared most chronic stress-induced alterations of metabolic and inflammatory factors. This interpretation accounts directly for the convergent behavioral and glycemic effects of 2 agents whose primary mechanisms, acute glycemic effects, and measured insulinotropic actions all differ.

Repeated acute stress-induced hyperglycemia may nonetheless be pathogenic in itself, with the subsequent hypoglycemic shift arising as a compensatory response to it. Whereas such a causal sequence is consistent with the glyburide arm, liraglutide improved behavior and restored the hyperglycemic response without reducing the acute stress-induced glycemic response, a result not explained by a simple causal sequence from repeated acute hyperglycemia to compensatory hypoglycemia. Distinguishing these possibilities will require selectively attenuating the acute stress-induced hyperglycemic excursion by a manipulation that does not itself act on insulin secretion, together with continuous rather than intermittent monitoring of glucose across the stress period. The aspects of glucose regulation shared by the two drugs therefore remain to be identified, and may include postprandial glucose excursions or the glucose dependence of insulin secretion, neither of which was measured here.

It should be noted that chronic stress significantly elevated circulating insulin levels in C57BL/6N mice, and produced a trend-level increase in BALB/c mice, which might appear to argue against the use of insulin-secreting agents in either strain. However, our aim was not to further enhance insulin secretion under basal, unstressed conditions or during behavioral testing, but rather to determine whether administration of these antidiabetic drugs before each of the 10 stress exposures could modify stress-induced glycemic responses and thereby prevent the subsequent development of behavioral deficits. Whether the hypoglycemic shift is itself beneficial or detrimental to behavior is a question distinct from what causes it, and is not resolved by the present data. Clinical studies report increased glucose uptake in specific brain regions of patients with depression, which is normalized by antidepressant treatment,^37–39^ raising the possibility that a lowering of systemic glucose availability could be beneficial.

Beyond peripheral glucose lowering, insulin may act directly in the brain. Insulin crosses the blood–brain barrier and supports synaptic function and plasticity, and deleting insulin receptors in mice produces depression-related behaviors.^40^^,^^41^ Enhanced insulin secretion induced by antidiabetic drugs could therefore help protect neural circuits from chronic stress-related dysfunction. Moreover, GLP-1 receptor agonists, including liraglutide, have additional direct central actions that could contribute to antidepressant-like effects, raising the need to disentangle their peripheral metabolic and central neural mechanisms.^42^^,^^43^ Glyburide may likewise act through mechanisms beyond insulin secretion, including potential anti-inflammatory effects.^44^

Two limitations of the present study should be acknowledged. First, all experiments were conducted exclusively in male mice. Given well-established sex differences in stress respsonsivity, glucose metabolism, and vulnerability to depression-related phenotypes, the generalizability of the present findings to females remains uncertain and warrants future investigation. Second, because the BALB/c data after chronic stress were obtained exclusively from animals that survived the stress protocol, the observed hypoglycemia should be interpreted as a finding in the surviving animals and should not necessarily be generalized as a characteristic response of the BALB/c strain. The high mortality also raises the possibility of survivorship bias in this group. This limitation does not affect our conclusion regarding the blunted hyperglycemic response observed in C57BL/6N and C57BL/6J mice, in which mortality during the chronic stress protocol was low.

In conclusion, our findings demonstrate that chronic stress induces a hypoglycemic shift accompanied by depression-related behaviors, and that the development of both can be prevented by antidiabetic drugs with distinct primary mechanisms. The mechanisms underlying this chronic stress-induced hypoglycemic shift, its interaction with genetically determined variability in acute stress-induced glycemic responses, and the extent to which altered glucose regulation contributes to stress-related neural dysfunction remain to be clarified. Nonetheless, our results provide experimental evidence for a close link between depression and T2D and suggest that glucose regulation is a candidate target for depressive symptoms in patients with diabetes. Because the drugs were administered before each of the 10 stress exposures and were discontinued before behavioral testing, the present study shows that they reduced the development of stress-induced behavioral changes rather than that they treated an already established phenotype; whether antidiabetic drugs are efficacious in a post-stress treatment design remains to be tested. With this caveat, the observation that antidiabetic drugs limited the development of depression-related behaviors in stressed mice without diabetes indicates that agents acting on glucose regulation merit further evaluation in stress-related disorders even in the absence of overt hyperglycemia.

## Supplementary Material

Qiu_SupplementaryFigures_260713_pyag046

## Data Availability

The datasets generated during and/or analyzed during the current study are available from the corresponding author on reasonable request.
