# Supplementary material for: Chronic social defeat stress induces a hypoglycemic shift and depression-related behaviors, both prevented by antidiabetic drugs
Source: Int J Neuropsychopharmacol. 2026 Aug 23;29(9):pyag046. doi: 10.1093/ijnp/pyag046 (PMC13557117; doi:10.1093/ijnp/pyag046)
Supplement: Supplementary file 1 [file qiu_supplementaryfigures_260713_pyag046.docx]

**Supplementary Fig. S1 Food intake and body weight during chronic stress in C57BL/6N and BALB/c mice**
(a) 3-h dark-phase food intake at Prestress, Day10, and Day11 of the chronic stress protocol in C57BL/6N (green, n = 5) and surviving BALB/c (blue, n = 3) mice. (b) Body weight of C57BL/6N mice (n = 5) from prestress through Day11 of chronic stress, shown as group mean (left) and individual animal trajectories (right). (c) Body weight of all BALB/c mice initially enrolled (n = 5), shown as group mean (left) and individual animal trajectories (right); the marked decline evident in the individual trajectories reflects two mice that subsequently died during the stress protocol. (d) Body weight of BALB/c mice that survived to the end of the protocol (n = 3), shown as group mean. Data are presented as mean ± SEM. ****P* < 0.001, and ns, not significant for Holm-Sidak's multiple comparisons test following a mixed-effects model (REML), comparing each time point with Prestress.

**Supplementary Fig. S2 Glyburide, but not liraglutide, increases circulating insulin**
(a) Serum insulin concentration in naive C57BL/6N mice 60 min after a single injection of vehicle, glyburide, or liraglutide. (b) Serum insulin concentration in BALB/c mice 60 min after a single injection of vehicle, glyburide, or liraglutide, in naive (gray) and chronically stressed (red) mice. Data are presented as mean ± SEM. *P < 0.05 for an unpaired t-test comparing vehicle and glyburide, following removal of one outlier from the vehicle group identified by Grubbs' test (a); #P < 0.1 for Holm-Sidak's multiple comparisons test comparing vehicle and glyburide, following a one-way ANOVA in which naive and chronically stressed mice were pooled within each drug group (b). In (a), n = 3 (after outlier removal), 4, and 4 for vehicle, glyburide, and liraglutide, respectively. In (b), n = 4, 4, and 4 for vehicle, glyburide, and liraglutide, respectively, in naive mice, and n = 3, 4, and 3 for vehicle, glyburide, and liraglutide, respectively, in chronically stressed mice.
